# Application of Fabric Phase Sorptive Extraction as a Green Method for the Analysis of 10 Anti-Diabetic Drugs in Environmental Water Samples

**DOI:** 10.3390/molecules29204834

**Published:** 2024-10-12

**Authors:** Augosto Misolas, Mohamad Sleiman, Vasilios Sakkas

**Affiliations:** 1 Department of Chemistry, School of Science, University of Ioannina, 451110 Ioannina, Greece; a.misolas@uoi.gr; 2 Institute of Chemistry of Clermont Ferrand, Sigma Clermont, UCA Campus Des Cezeaux, Université Clermont Auvergne, Clermont Auvergne INP, CNRS, ICCF, F-63000 Clermont-Ferrand, France; mohamad.sleiman@sigma-clermont.fr

**Keywords:** fabric phase sorptive extraction, anti-diabetic drugs, environmental water

## Abstract

Due to the increased prevalence of diabetes, the consumption of anti-diabetic drugs for its treatment has likewise increased. Metformin is an anti-diabetic drug that is commonly prescribed for patients with type 2 diabetes and has been frequently detected in surface water and wastewaters, thus representing an emerging contaminant. Metformin can be prescribed in combination with other classes of anti-diabetic drugs; however, these drugs are not sufficiently investigated in environmental samples. Fabric phase sorptive extraction (FPSE) has emerged as a simple and green method for the extraction of analytes in environmental samples. In this study, FPSE coupled with a high-performance liquid chromatography diode array detector (HPLC-DAD) was employed for the simultaneous analysis of different classes of anti-diabetic drugs (metformin, dapagliflozin, liraglutide, pioglitazone, gliclazide, glimepiride, glargine, repaglinide, sitagliptin, and vildagliptin) in environmental water samples. Four different fabric membranes were synthesized but the microfiber glass filter coated with sol-gel polyethylene glycol (PEG 300) was observed to be the best FPSE membrane. The parameters affecting the FPSE process were optimized using a combination of one-factor-at-a-time processes and the design of experiments. The FPSE was evaluated as a green extraction method, based on green sample preparation criteria. The FPSE-HPLC-DAD method achieved acceptable validation results and was applied for the simultaneous analysis of anti-diabetic drugs in surface and wastewater samples. Glimepiride was detected below the quantification limit in both lake and river water samples. Dapagliflozin, liraglutide, and glimepiride were detected at 69.0 ± 1.0 μg·L^−1^, 71.9 ± 0.4 μg·L^−1^, and 93.9 ± 1.3 μg·L^−1^, respectively, in the city wastewater influent. Dapagliflozin and glimepiride were still detected below the quantification limit in city wastewater effluent. For the hospital wastewater influent, metformin and glimepiride were detected at 1158 ± 21 μg·L^−1^ and 28 ± 0.8 μg·L^−1^, respectively, while only metformin (392.6 ± 7.7 μg·L^−1^) was detected in hospital wastewater effluent.

## 1. Introduction

Diabetes mellitus is a persistent metabolic disorder characterized by increased levels of blood glucose due to a deficiency in insulin secretion [1]. According to the International Diabetes Federation report in 2021, the number of adults (aged 20–79 years old) with diabetes in Europe was estimated at 67 million, corresponding to 9.2% of the whole population, and this figure is expected to increase to 9.8% by 2030 and 10.4% by 2045. In Greece, the diabetes prevalence was estimated at 9.6% [2]. Type 2 diabetes mellitus (T2DM) or non-insulin-dependent diabetes is the most common form, accounting for around 85–90% of all diagnosed cases [1]. Metformin, the only drug belonging to the biguanide class of anti-diabetic drugs, is the first-line treatment for T2DM by reducing the glucose level in the blood. Due to its polarity and not being completely metabolized, the drug has frequently been detected in environmental waters [3,4]; it is considered one of the emerging contaminants and may cause environmental toxicity at very low concentrations [5].

Metformin is prescribed alone or in combination with other classes of anti-diabetic drugs like dapagliflozin (sodium-glucose cotransporter type 2 inhibitor), liraglutide (glucagon-like peptide-1 agonist), pioglitazone (thiazolidinedione), gliclazide and glimepiride (sulfonylureas), repaglinide (meglitinide), glargine (insulin), and vildagliptin and sitagliptin (dipeptidyl peptidase-4 inhibitors). They all have different mechanisms of action to either increase insulin secretion or reduce glucose production [6]. In Athens, the consumption of anti-diabetic drugs was estimated at 13,014 g·day^−1^ in 2020 using wastewater-based epidemiology [7]. Aside from metformin and its metabolite, guanyl urea, different classes of anti-diabetic drugs have been identified in water and wastewater [8,9].

For the determination of anti-diabetic drugs in various matrices, different analytical techniques have been employed, like high-performance liquid chromatography (HPLC) coupled with ultraviolet-visible [10], diode array (DAD) [11], or photodiode array (PDA) [12] detection. To increase the detection sensitivity, a liquid chromatography mass spectrometry (MS) detector is utilized [13,14]. Electrochemical assays have also been reported [15]. Prior to instrumental analysis, anti-diabetic drugs must be extracted from the sample matrix using different methods such as liquid–liquid extraction (LLE) [16] and solid-phase extraction (SPE) [17].

Vortex-assisted dispersive liquid–liquid microextraction (VA-DLLME) has been applied for the extraction of anti-diabetic drugs from river water samples [18], while solid phase extraction (SPE) has been used on surface and wastewater samples [19,20,21]. A quick, easy, cheap, effective, and rugged method of safe-syringe filter-based micro-solid phase extraction (QuEChERS SF-μSPE) has previously been proposed as a green extraction method [22]. Recently, fabric phase sorptive extraction (FPSE) has also been applied for the extraction of three anti-diabetic drugs in human plasma [23].

FPSE is an extraction method introduced in 2014 by Kabir and Furton to overcome the challenges found in other sorptive extraction techniques, such as low sorbent capacity and a long extraction time [24,25]. FPSE is an equilibrium-based microextraction technique exploiting sol-gel technology. Sol-gel is formed when a liquid colloidal solution (sol) from the hydrolysis of a precursor undergoes polycondensation and forms a 3D network of a solid matrix (gel) with an inorganic/organic polymer. A sorbent material is prepared by thinly coating a hydrophilic, neutral, or hydrophobic substrate (fabric) with the sol-gel. A variety of sorbent materials can be prepared by using different combinations comprising a substrate, one or more inorganically/organically modified sol-gel precursors, the sol-gel active inorganic/organic polymer, solvent, catalyst, and water for hydrolysis [26]. FPSE is a two-step extraction method utilizing the minimum amount of organic solvent, satisfying the principles of green analytical chemistry [27].

FPSE has been applied in the extraction of different contaminants in environmental samples [28,29,30,31,32], but, to the best of our knowledge, the potential of this method for the extraction of anti-diabetic drugs in water has not yet been investigated. This study proposes an easy and green analytical method for the extraction of different classes of anti-diabetic drugs (metformin, dapagliflozin, liraglutide, pioglitazone, gliclazide, glimepiride, glargine, repaglinide, sitagliptin, and vildagliptin) from environmental water samples by applying FPSE combined with HPLC-DAD. The significant parameters affecting the extraction efficiency were determined and optimized by employing a combination of the one-factor-at-a-time approach, a Box–Behnken design, and the response surface methodology. The greenness of the proposed method was evaluated using metric tools based on the principles of green analytical chemistry and compared them with other reported methods. Lastly, the FPSE-HPLC-DAD method was validated and applied for the analysis of real environmental water samples (both surface water and wastewater).

## 2. Results and Discussion

### 2.1. Preliminary FPSE Experiments

To maximize the extraction efficiency of the target analytes from water, the FPSE conditions were optimized using univariate and multivariate analyses. Parameters such as the type of fabric substrate, type of sol-gel coating, agitation mode, sample pH, and desorption solvent were initially determined via a series of experiments.

Firstly, the fabric substrate and the sol-gel coating were selected. The substrate surface chemistry and the polarity of the sol-gel coating affect the selectivity and extraction efficiency of an FPSE membrane. Since the polarity of the analytes varies from highly polar to less polar (Table S1 and Figure S1), two sol-gel coatings with different polarities were assessed.

In this study, different combinations of fabric and sol-gel coating were evaluated: a sol-gel polyethylene glycol-coated microfiber glass filter (GF-PEG), a sol-gel poly(ethylene glycol)-block-poly(propylene glycol)-block-poly(ethylene glycol)-coated microfiber glass filter (GF-PEG-PPG-PEG), a sol-gel PEG-coated cellulose filter (CF-PEG), and a sol-gel PEG-PPG-PEG-coated cellulose filter (CF-PEG-PPG-PEG). A solution of 1000 μg·L^−1^ of anti-diabetic drugs, made using Milli-Q water, was prepared to determine the adsorption efficiency of the different FPSE membranes. The FPSE conditions of a 1-mL sample volume and 30 min of extraction time using vortex shaking were selected, based on our previous laboratory experience [33]. The adsorption efficiency (%) was calculated using Equation (1):

(1) $$\mathrm{Adsorption}\;\mathrm{efficiency}\;\left(\%\right)=\frac{\left({\mathrm{PA}}_{\mathrm{before}}-{\;\mathrm{PA}}_{\mathrm{after}}\right)}{{\mathrm{PA}}_{\mathrm{before}}}\times 100$$

where *PA_before_* and *PA_after_* are the peak areas of the analyte before and after the FPSE.

**Figure 1:**
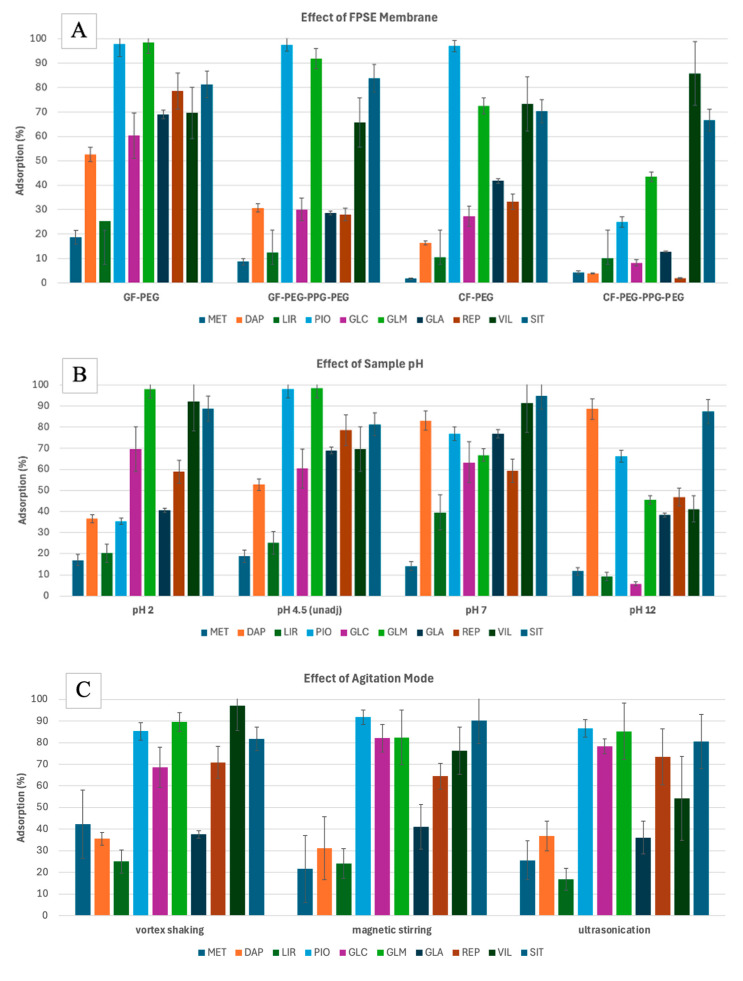

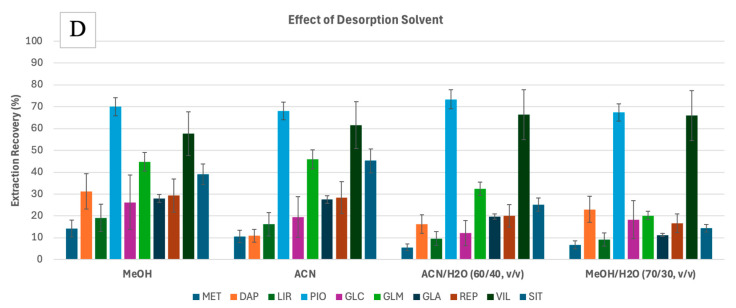
Preliminary experiments for the FPSE of 10 anti-diabetic drugs drawn from water, assessing the effect of FPSE membrane type (**A**), pH of the sample (**B**), and agitation mode (**C**) on the adsorption efficiencies, and the effect of the desorption solvent (**D**) on the extraction recoveries. Error bars represent the standard deviation of triplicates. Analytes: MET—metformin, DAP—dapagliflozin, LIR—liraglutide, PIO—pioglitazone, GLC—gliclazide, GLM—glimepiride, GLA—glargine, REP—repaglinide, VIL—vildagliptin, SIT—sitagliptin.

Based on the results (Figure 1A), the GF-PEG membrane had the highest adsorption efficiencies. GF is a neutral substrate, which has previously been demonstrated to be effective for the extraction of less polar analytes [27]. PEG (polyethylene glycol), on the other hand, is a polar polymer that facilitates the extraction of polar analytes. The combination of GF and PEG likely creates a versatile extraction medium that is capable of interacting with analytes across a wide range of polarities, which explains the superior performance that was observed. This synergy between the two materials allows for the efficient extraction of both more polar and less polar compounds. Hence, the GF-PEG FPSE membrane was selected for further experiments.

Using the GF-PEG membrane, the adsorption efficiencies (%) at different sample pH were measured (Figure 1B). Since the analytes have different pKa values (Table S1), they will behave differently at different pH values. pH adjustment was used to force the analytes to remain in their neutral form to maximize the extraction efficiencies. Most analytes are weak bases; as such, they are neutral when pH is at pKa + 2 [33]. However, this trend was not observed for all the analytes. For example, analytes such as PIO, GLC, GLM, and REP, which had pKa values ranging from 5.2 to 6.2, showed higher adsorption at pH 4.5. In contrast, analytes like SIT, VIL, and LIR, with pKa values between 7.7 and 9.5, exhibited higher adsorption at pH 7. MET did not show significant changes in adsorption with varying pH, likely due to its two pKa values. DAP, with a pKa of 12.6, exhibited maximized adsorption at pH 12. Despite its high pKa, DAP is a non-ionizable molecule with a solubility that remains largely unaffected by pH changes [34]. Its slight solubility in aqueous solutions likely explains its superior extraction efficiency at pH 7 and 12 compared to other analytes. Limited exposure to highly acidic/basic environments does not impact the performance of silica- or cellulose-based sorbents [26]. Additionally, other properties, such as the analytes’ logKow values, may have played a more significant role in their extraction performance. There was no significant difference in the average adsorption of all target analytes at pH 4.5 or pH 7. Hence, the sample pH was not adjusted in the following experiments. In this pH range (around pH 6), maximum extraction was also observed for SIT, GLC, MET, and REP during micro-solid phase extraction [22].

The FPSE equilibrium can be attained more quickly when the analyte diffusion in the matrix is increased by applying external stimuli such as agitation. In this study, different modes of agitation were assessed. Testing the FPSE of 1000 μg·L^−1^ solution (1 mL) was performed using vortex shaking (10 Hz), magnetic stirring (300 rpm), and ultrasonication (20 kHz) for 30 min. Different adsorption efficiencies (%) were obtained for each analyte with different agitation modes (Figure 1C). However, when calculating the mean desorption (%) for each mode, the vortex shaking performance had the highest level, followed by magnetic stirring, and then ultrasonication. Hence, vortex shaking was selected.

Finally, the choice of organic solvent for the FPSE desorption was determined using 0.1 mL of organic solvent. Different polar organic solvents, such as ACN and MeOH, at different percentages in water were used. The extraction recoveries (%) using the organic solvents were determined using Equation (2):

(2) $$\mathrm{Extraction}\;\mathrm{Recovery}\;\left(\%\right)=\frac{\left({\mathrm{PA}}_{\mathrm{elution}}\right)\left(V_{\mathrm{elution}}\right)}{\left({\mathrm{PA}}_{\mathrm{after}}\right)\left(V_{\mathrm{sample}}\right)}\times 100$$

where *PA_elution_* and *PA_after_* are the peak areas of the analyte after elution and before FPSE, respectively, and *V_elution_* and *V_sample_* are the volume of desorption solvent and the sample, respectively.

Polar organic solvents were used since the target analytes are moderately to highly polar. Based on the results (Figure 1D), lower extraction recoveries were obtained when organic solvents were mixed with H_2_O. Conversely, there was no significant differences in the recovery figures when ACN or MeOH was used. To allow better compatibility with the mobile phase of HPLC analysis, ACN was selected initially.

### 2.2. Experimental Design and Optimization

To further establish the optimized conditions for the FPSE process, a design of experiments was conducted for the FPSE adsorption and desorption processes. A Box–Behnken design (BBD) with 15 experiments each was employed for each process. For the adsorption, sample volume (1, 5, and 10 mL), ionic strength (0, 1, and 2% NaCl), and adsorption time (15, 30, and 45 min) were used as independent variables while the mean adsorption efficiency (%) was used as the dependent variable. In contrast, for the desorption, solvent (0, 50, and 100% of ACN in ACN/MeOH mix), elution volume (0.2, 0.6, 1 mL), and elution time (10, 20, and 30 min), were used as the independent and mean extraction recovery (%) dependent variables, respectively. These factors directly influence the FPSE process, while temperature is typically not optimized [28]. All extraction procedures were performed at room temperature. The results of the analysis of variance (ANOVA) and response surface methodology (RSM) were obtained using Statgraphics 19 (Statgraphics Technologies Inc., The Plains, VA, USA).

**Figure 2:**
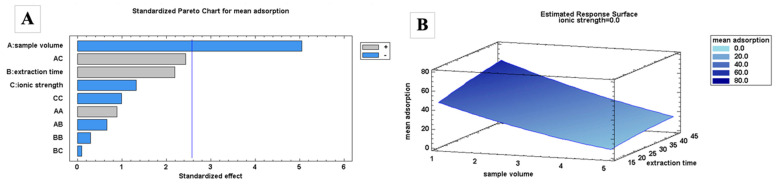

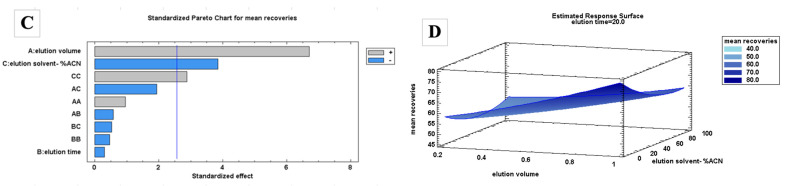
Pareto charts and response surface plots for FPSE adsorption (**A**,**B**) and desorption (**C**,**D**) using a Box–Behnken design.

Regarding the adsorption process, the BBD quadratic model was able to explain 89.0% of the variability in the mean adsorption efficiencies. The sample volume was significant at a 95.0% confidence level according to the ANOVA and Pareto chart (Table 1 and Figure 2A). Since the FPSE membrane is fixed, with a round shape of 1 cm in diameter, its extraction efficiency depends on the sample volume. Based on the results, the extraction efficiency can be maximized using a lower sample volume. A higher sample volume may be required when using larger sizes of FPSE membranes. The effects from the interaction of the different factors were insignificant.

For the desorption process, the BBD quadratic model was able to explain 93.6% of the variability in terms of the mean extraction recoveries. The volume of the elution solvent and the %ACN were significant at a 95.0% confidence level (Table 1 and Figure 2C). Maximum recoveries can be obtained using a higher volume of organic solvent. The use of a higher volume of solvent exposes the whole FPSE membrane with the solvent, leading to better back-extraction. The desorption was better when using a lower percentage of ACN, but the effect of the interaction of this factor was observed to be significant. Based on the chromatograms, lowering the percentage of ACN affects the peak areas of the polar analytes, leading to broader peaks and coelutions (Figure S2). Therefore, 100% ACN is preferred for more accurate quantitative analyses.

Based on the RSM (Figure 2B,D), the adsorption efficiencies can be maximized when a lower sample volume, no ionic strength, and a longer extraction time are used. Ionic strength enables the polar analytes to move toward the surface of the water, minimizing their interaction with the sorbent. Increasing the ionic strength also increases the viscosity of the sample, which negatively affects the extraction kinetics. A longer extraction time means that a longer time is needed to reach equilibrium. In contrast, higher extraction recoveries can be obtained using a larger volume of organic solvent. However, a lower volume of solvent is preferable to increase the preconcentration factor. Although extraction recoveries are maximized at a lower %ACN, as mentioned previously, lowering the %ACN results in broader peaks and coelutions. There was no significant difference recorded in recovery values when the desorption time was increased.

Hence, optimized conditions for the FPSE of anti-diabetic drugs in water are the following: GF-PEG, 1 mL sample volume, no pH adjustment, 0% ionic strength, 45 min extraction time using vortex shaking, 0.1 mL of 100% ACN as elution solvent, and 10 min elution time.

Using the optimized conditions, the extraction recoveries (ER %) were 13.3 ± 0.6% for MET, 64.0 ± 5.7% for DAP, 25.5 ± 1.5% for LIR, 73.4 ± 3.4% for PIO, 45.5 ± 1.1% for GLC, 79.2 ± 4.1% for GLM, 35.0 ± 1.9% for GLA, 71.6 ± 4.7% for REP, 38.6 ± 0.4% for SIT, and 40.0 ± 2.7% for VIL. Higher ER% values were obtained for less polar analytes than for more polar analytes. Due to MET’s high polarity and water solubility, equilibrium between the sample matrix and the FPSE membrane may not be so easily achieved within the given extraction time. A longer extraction time could potentially improve its extraction efficiency. An ion exchange sorbent may also be more suitable for capturing MET, given its polar and ionic nature compared to PEG.

Mean extraction recoveries can be improved by using more polar organic polymers for the synthesis of the FPSE membranes. Furthermore, a magnet can be integrated into the FPSE membrane to have precise control when agitating the sample, leading to better diffusion of the target analytes in the sample matrix [35]. The preconcentration factor for all analytes was 10. The enrichment factor (EF) is calculated as the ratio between the concentration of the analyte in the eluent and the concentration of the analyte before the extraction. Hence, the EF values for the analytes were found to be 1.3 (MET), 2.6 (LIR), 3.5 (GLA), 3.9 (SIT), 4.0 (VIL), 4.6 (GLC), 6.4 (DAP), 7.2 (REP), 7.3 (PIO), and 7.9 (GLM). The analytes can be ranked based on their logKow values and hydrophilicity, as follows: LIR < MET < SIT < VIL < GLC < DAP < PIO < GLM < REP. In general, ER% and EF tended to increase with higher logKow values. While the logKow data for GLA were unavailable (according to our knowledge), its high molecular weight may have contributed to better adsorption on the membrane surface, resulting in higher extraction efficiency compared to MET and LIR.

### 2.3. Analytical Performance of the FPSE-HPLC-DAD Method

The optimized extraction conditions were applied prior to the HPLC-DAD analysis regarding anti-diabetic drugs. Subsequently, the method was validated according to the ISO 17025 guidelines [36]. Linearity was obtained for each analyte by analyzing five calibration solutions with increasing concentrations. The coefficients of determination were above 0.990, except for SIT and VIL, which did not provide good calibration results. The limit of quantification (LOQ) and limit of detection (LOD) for each anti-diabetic drug were calculated as the minimum analyte concentrations, for which the signal-to-noise (S/N) ratios were 10 and 3, respectively. The LOQs and LODs were in the range of 6.7–110 μg·L^−1^ and 2–34.6 μg·L^−1^, respectively. The intra-day and inter-day precision values are expressed as the percentages of the relative standard deviation of spiked solutions (50 μg·L^−1^ and 300 μg·L^−1^) analyzed in triplicate after 1 day and 3 days, respectively. The %RSD were all below 12%. The relative recoveries (RR %) were determined by spiking 300 μg·L^−1^ of analytes into Milli-Q water. The RR % values were in the range of 94.8–103.1%. The figures of merit for each analyte are presented in Table S2.

### 2.4. Application in Real Water Samples

The feasibility of applying the proposed FPSE-HPLC-DAD method to real environmental water samples was investigated. Anti-diabetic drugs were quantified in surface water (lake and river) and wastewater (influent and effluent) samples. A total of six water samples were analyzed (Table S3). Each analysis was performed in replicates under the optimized conditions. GLM was detected in both lake and river water samples, although levels were below the LOQ. DAP, LIR, and GLM were all detected in the city WWTP influent. DAP and GLM were detected in the city WWTP effluent, but levels were below the LOQ. For the hospital WWTP influent, MET and GLM were detected, while only MET was detected in the hospital WWTP effluent. The hospital wastewater passes directly to the city’s WWTP for further treatment. The significant decrease in the concentrations of the target analytes from influent to effluent may signify the effectiveness of water treatment, although contaminant removal can still be improved prior to its discharge to water bodies. A previous study has reported the detection of MET in the city and hospital WWTP, where maximum concentrations were found to be 249 ng·L^−1^ and 1167 ng·L^−1^, and 23 ng·L^−1^ and 26 ng·L^−1^, for the influents and effluents, respectively [37]. A significant increase (compared to almost 10 years ago) in the concentration of MET in the wastewater samples may be due to the increased prevalence of diabetes and the subsequent increase in the use and misuse of the anti-diabetic drug.

**Figure 3:**
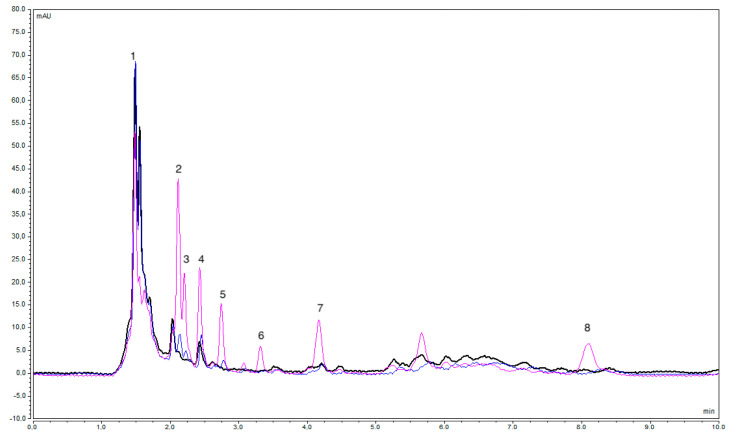
Overlay of HPLC chromatograms for the undiluted unspiked hospital WWTP influent (black), spiked with 50 μg·L^−1^ (blue), and spiked with 300 μg·L^−1^ (pink), after application (without dilution) of the proposed FPSE-HPLC under optimized conditions at a 224 nm wavelength. Peaks: 1—MET, 2—DAP, 3—LIR, 4—GLA, 5—PIO, 6—GLC, 7—GLM, and 8—REP.

The accuracy of the proposed method was checked by determining the concentration of the target analytes in unspiked and spiked real water samples. The relative recovery (RR %) was calculated by dividing the difference in concentrations between spiked and unspiked water samples by the spiked concentration. The RR % was assessed at two concentration levels, 50 μg·L^−1^ and 300 μg·L^−1^. Depending on the sample matrix, the RR % value varied (Table S3). The maximum relative recoveries of the target analytes across the different water samples were in the range of 59.2% (GLC) to 111.9% (MET). The RR % was affected by some matrix effects, as assessed. The matrix effect (ME %) was calculated by dividing the difference in peak areas between the spiked and unspiked samples by the peak area of the target analyte in the spiked Milli-Q water [22]. Significant matrix effects were noted in the analysis of wastewater samples. Across the real water samples, the ME % was in the range of 5.4% to 112.8%. Since the FPSE did not require filtration prior to the extraction process, the presence of some solids may have interfered in the extraction of the analytes. Furthermore, unknown compounds were eluted at the same retention time used with the target compounds, as in the case of GLA in the hospital WWTP influent (Figure 3). This was confirmed by comparing the UV-Vis spectra with the standard solutions. The chromatograms of the other water samples are displayed in Figure S3.

### 2.5. Evaluation of Method’s Greenness

**Figure 4:**
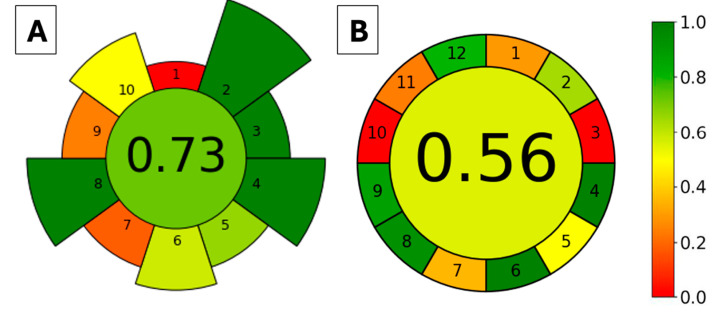
Evaluation of the method’s greenness based on the AGREEprep (**A**) and AGREE (**B**) metric tools. AGREEprep criteria: 1—sample preparation, 2—solvents, 3—sustainable materials, 4—waste, 5—sample size, 6—sample throughput, 7—sample steps and automation, 8—energy consumption, 9—post-sample preparation, and 10—operator safety. AGREE criteria: 1—sample treatment, 2—sample size, 3—device positioning, 4—sample preparation steps, 5—automation and miniaturization, 6—derivatization, 7—waste generation, 8—analysis throughput, 9—energy consumption, 10—source of reagents, 11—toxicity, and 12—operator safety.

The greenness of the proposed method was evaluated using two approaches, AGREEprep [38] and AGREE [39], for the extraction method (FPSE) and the whole analytical method (FPSE-HPLC-DAD), respectively. The AGREEprep metric tool evaluates the sample preparation method according to 10 criteria that are based on the 10 principles of green sample preparation. In contrast, the AGREE metric tool assesses the whole analytical procedure according to the 12 principles of green analytical chemistry; hence, there are 12 evaluation categories. For both metrics, each criterion is assigned with a score in the range of 0.0–1.0, with 1.0 indicating the highest greenness score. The scores are then represented using a color gradient from red to green. The proposed method was evaluated, and the resulting pictograms are depicted in Figure 4A and Figure 4B for AGREEprep and AGREE, respectively. In the AGREEprep graph, the red color (criterion 1) was due to the FPSE being performed with ex situ sample preparation. The orange color of criteria 7 and 9 was due to the non-automation of FPSE and LC, respectively, as a post-extraction analytical technique. Still, the FPSE method attained an overall score of 0.73, which indicates that the extraction method has acceptable green characteristics. For the AGREE graph, red colors are used for principles 3 (ex situ sample preparation) and 10 (that none of the reagents are bio-based). Principles 1, 7, and 11 are shown in an orange color since the proposed method has an external sample pre-treatment, generates 12.1 mL of liquid waste (0.1 mL desorption solvent and 1.2 mL·min^−1^ flow rate of mobile phase for 10 min), and uses 7.3 mL of toxic reagent (ACN, 0.1 mL as desorption solvent and 7.2 mL as mobile phase). The overall AGREE score of the FPSE-HPLC-DAD method was 0.56, which still represents acceptable greenness.

### 2.6. Comparison with Published Methods

To further highlight the greenness of FPSE as an extraction method for the analysis of anti-diabetic drugs in environmental water samples, the proposed method was compared with other published methods (Table 2) according to the AGREEprep criteria. All the published methods were performed ex situ. The FPSE requires the least amount of sample volume. SPE-based methods require several steps (pH adjustments, cartridge conditioning, sample loading, washing, and eluting) resulting in the use of larger amounts of toxic organic solvents. The FPSE method only requires organic solvent at a minimum volume for the elution of extracted analytes. Hence, a larger amount of waste is generated when using the SPE method compared with FPSE and DLLME. Although the FPSE method requires a longer extraction time, the number of analytes extracted was maximized. Using LCMS as a post-extraction analytical technique results in better sensitivities but the energy consumption is higher than with HPLC-UV/DAD. In terms of sample analysis throughput, the FPSE-based method had the highest throughput. Overall, FPSE represents a greener method for the extraction of anti-diabetic drugs from environmental water samples. FPSE offers several advantages over other microextraction techniques. It features a simple and streamlined workflow with only 2 to 3 steps, requires a low volume of organic solvents, and provides a larger contact surface area, allowing for faster extraction equilibrium. Additionally, the sorbent is easy to prepare, and various combinations of fabrics and polymers can be synthesized based on the properties of the analytes, offering flexibility and adaptability.

## 3. Materials and Methods

### 3.1. Chemicals and Reagents

The fabric substrates used in the experiments were microfiber glass filters (GF) with a diameter of 110 mm and cellulose filters (CF) with a diameter of 125 mm, sourced from Whatman (Boston, MA, USA). The organic polymers polyethylene glycol (PEG 300) and poly(ethylene glycol)-block-poly(propylene glycol)-block-poly(ethylene glycol) (PEG-PPG-PEG 5800) were purchased from Sigma-Aldrich (Athens). Organic solvents such as dichloromethane (DCM), acetonitrile (ACN), acetone, and methanol (MeOH) were purchased from Merck (Darmstadt, Germany). Likewise, hydrochloric acid (HCl), sodium hydroxide (NaOH), trifluoroacetic acid (TFA), and methyl trimethoxysilane (MTMS) were purchased from Merck (Darmstadt, Germany).

During the method development, high-purity standard solutions of the active ingredients were used to ensure the precision and accuracy needed for calibration and validation of the analytical method. The physicochemical characteristics of the drugs are presented in Table S1, and their chemical structures are shown in Figure S1. To ensure their application to real samples, simulating real-world conditions and ensuring the method’s applicability, pharmaceutical formulations (such as tablets or other dosage forms) were used as the source of the active ingredient (Table S1). These real drug formulations were employed to account for the presence of excipients and other ingredients commonly found in commercial products, which could potentially affect the method’s performance. The analytes were dissolved in methanol and filtered to obtain the following stock concentrations: 1000 mg·L^−1^ MET, 200 mg·L^−1^ DAP, 300 mg·L^−1^ PIO, 600 mg·L^−1^ GLC, 40 mg·L^−1^ GLM, 100 mg·L^−1^ REP, 1000 mg·L^−1^ SIT, and 1000 mg·L^−1^ VIL. Stock solutions of GLA (3640 mg·L^−1^) and LIR (6000 mg·L^−1^) were purchased in liquid form. Working solutions in Milli-Q water were prepared daily.

### 3.2. Collection of Environmental Water Samples

Conventional water sampling (with a 2 L composite water sampler made of ultra-high-molecular-weight polyethylene (UHMW), PVC, and stainless steel hardware) was carried out for the targeted analysis of anti-diabetic drugs. Different environmental water samples were collected from different locations. Surface water samples were collected from the Arachtos River and Pamvotis Lake. Wastewater samples were collected from the hospital and the city wastewater treatment plant (WWTP).

Grab water samples were collected in clean 1 L amber glass bottles. Collected samples were transported immediately to the laboratory and refrigerated at 5 °C until extraction and analysis. Six samples (river, lake, city WWTP influent and effluent, and hospital WWTP influent and effluent) were extracted within 24 hr after collection.

### 3.3. HPLC-DAD Analysis

A Thermo Fisher Scientific UltiMate 3000 HPLC system (Waltham, MA, USA), equipped with a binary solvent manager, WPS−3000SL autosampler, column manager, and diode array detector (DAD), was used for the separation of anti-diabetic drugs. Different chromatographic parameters such as mobile phase compositions, elution type (gradient versus isocratic), flow rate, column temperature, and detection wavelength were tested by analyzing a standard solution containing a mixture of all the target analytes until the optimized HPLC conditions were attained.

A 20 μL sample was injected onto a Hypersil GOLD C18 column (Thermo Scientific, Waltham, MA, USA) with dimensions of 150 × 4.6 mm and a particle size of 5 μm, maintained at 25 °C.

An isocratic elution using a mobile phase consisting of ACN (A) and 0.05 M phosphate buffer pH 3.79 (B) at a 1.2 mL·min^−1^ flow rate for 10 min was used for the separation of analytes. For the qualitative and quantitative analysis, the DAD was set at 236 nm for MET, 224 nm for DAP, PIO, GLC, and GLM, 272 nm for LIR, and 212 nm for VIL, GLA, SIT, and REP. Full spectral scans were also obtained from 200 to 400 nm. The Chromeleon 7 version 7.2.2.6394 (Thermo Fisher, Waltham, MA, USA) software was used for the HPLC data analysis. Examples of chromatograms using the unoptimized and optimized HPLC conditions are shown in Figures S4 and S5 while the resolution and theoretical plates are presented in Table S4.

### 3.4. Preparation of Sol-Gel Coated FPSE Membrane

#### 3.4.1. Pretreatment of Fabrics

The fabric substrates were pretreated to remove contaminants from the manufacturing process or those that may have accumulated over time, and to activate their surfaces and expose the hydroxyl or silanol groups for the sol-gel coating. Briefly, the CF was soaked in deionized water under sonication for 15 min and then washed with plenty of deionized water. The fabric was immersed in 1.0 M NaOH and sonicated for 1 h, then immersed in 0.1 M HCl for 1 h. After each acid/base treatment, the fabric was washed with copious amounts of deionized water. The fabric was then dried in ambient air for 24 h at room temperature. The same procedure was applied for the pretreatment of GF.

#### 3.4.2. Sol-Gel Coating of the Fabrics

The pretreated fabrics were coated with two types of sol-gel, prepared using two different polymers: PEG and PEG-PPG-PEG. The preparation of the sol-gel solution was as described previously in our earlier study, with some modifications [33]. Briefly, the sol-gel solution was prepared by dissolving 5 g of organic polymer, 5 mL of MTMS as a sol-gel precursor, 2 mL of 95% TFA as a catalyst, and 10 mL of a mixture of acetone and DCM (50/50 *v*/*v*) as the organic solvent. The molar ratio of 0.5:0.2:0.5:1.0 was maintained, corresponding with the organic polymer, precursor, catalyst, and organic solvent, respectively. The mixture was vortexed for 3 min, then centrifuged for 5 min at 5000 rpm, and finally, the clear supernatant was transferred to a clean reaction flask.

For the sol-gel coating, the pretreated fabric was soaked in the supernatant to form a three-dimensional network of sol-gel on the surface of the fabric. The fabric was kept immersed for 4 h. The coated fabric was then removed from the reaction vessel and dried in a desiccator overnight to evaporate the solvents. For the removal of unreacted and unbonded coating mixture, the sol-gel-coated fabric was immersed in a copious amount of organic solvent (acetone:DCM, 50/50, *v*/*v*) under sonication for 30 min. The dried sol-gel-coated fabric was then cut into smaller circular shapes with a diameter of 1 cm and kept in a sealed container until use.

In the end, four different types of FPSE membrane were prepared: sol-gel PEG-coated CF (CF-PEG), sol-gel PEG-PPG-PEG-coated CF (CF-PEG-PPG-PEG), sol-gel PEG-coated GF (GF-PEG), and sol-gel PEG-PPG-PEG-coated GF (GF-PEG-PPG-PEG). The membranes were characterized similarly to those in our previous study [33] and the scanning electron microscopy (SEM) images of PEG-coated GF are presented in Figure S6.

#### 3.4.3. Fabric Phase Sorptive Extraction Procedure

Firstly, the FPSE membrane was cleaned and activated by soaking it with 2 mL of MeOH:ACN (50:50, *v*/*v*) for 5 min. Next, the membrane was immersed in 2 mL of Milli-Q water to remove any residual organic solvent and then air-dried. The clean FPSE membrane was placed into a vial containing a predetermined volume of aqueous sample. The vial then underwent vortex shaking to increase the diffusion of the target analytes throughout the sample. After the predetermined extraction time, the FPSE membrane was removed from the vial and transferred to another clean vial. The target analytes were eluted using a sufficient volume of organic solvent. The eluted analytes were collected using a Pasteur pipette and transferred to an HPLC autosampler vial.

After a series of experiments and optimization using Milli-Q water spiked with 1000 μg·L^−1^ of all target analytes, the FPSE’s optimized conditions were determined to be a 1 mL sample, no pH and ionic strength adjustments, 45 min of vortex shaking, 0.1 mL ACN elution solvent, and 10 min of elution time.

## 4. Conclusions

In the current study, FPSE coupled with HPLC-DAD was applied for the first time to achieve the simultaneous extraction from and analysis of different classes of anti-diabetic drugs in environmental water samples. The FPSE membranes were synthesized using sustainable materials. The optimized FPSE conditions were obtained after univariate and multivariate optimizations. Extraction recoveries could be improved by synthesizing a more polar FPSE membrane or by the integration of a magnet for precise agitation of the samples. Compared with other extraction methods, the FPSE better satisfied the principles of green sample preparation according to the AGREEprep metric. Coupled with HPLC-DAD, the method has been validated and produced satisfactory results. The detection and quantification limits work within parameters that are good enough for the quantification of anti-diabetic drugs in water samples. Higher sensitivities could be achieved if LCMS is employed. The intra-day and inter-day precision values, shown as %RSD, were below 12%. The relative recoveries obtained were in the range of 94.8–103.1%. Finally, the FPSE-HPLC-DAD method has been applied for the analysis of anti-diabetic drugs in real water samples, wherein metformin was detected at high levels in hospital wastewater samples. The technique could be further improved to minimize matrix effects and increase relative recovery rates in the analysis of wastewater samples.

## Figures and Tables

**Table 1:** Results of the ANOVA in a Box–Behnken design for FPSE adsorption and desorption.

ADSORPTION					
Factor	SS	df	MS	*F*-ratio	*p*-Value
A: sample volume	1381.15	1	1381.15	25.44	0.0040
B: extraction time	259.38	1	259.38	4.78	0.0805
C: ionic strength	94.08	1	94.08	1.73	0.2451
AA	42.30	1	42.30	0.78	0.4178
AB	23.68	1	23.68	0.44	0.5381
AC	322.61	1	322.61	5.94	0.0588
BB	4.57	1	4.57	0.08	0.7832
BC	0.44	1	0.44	0.01	0.9318
CC	53.11	1	53.12	0.98	0.3680
**DESORPTION**					
**Factor**	**SS**	**df**	**MS**	***F*-ratio**	***p*-Value**
A: elution volume	430.80	1	430.80	44.94	0.0011
B: elution time	0.84	1	0.84	0.09	0.7791
C: elution solvent—%ACN	141.74	1	141.74	14.79	0.0121
AA	8.78	1	8.78	0.92	0.3824
AB	3.11	1	3.11	0.32	0.5934
AC	35.78	1	35.78	3.73	0.1112
BB	2.02	1	2.02	0.21	0.6653
BC	2.63	1	2.63	0.28	0.6223
CC	79.63	1	79.63	8.31	0.0345

**Table 2:** Comparison of the developed method with other published methods on the analysis of anti-diabetic drugs in surface and wastewater.

Sample Volume, mL	Analytes	Extraction Method	Extraction Time, min	Extraction Solvent/Volume, mL	Analytical Technique	Flow Rate (mL· min^−1^)	Analysis Time, min	LOD/LOD Range (μg·L^−1^)	Ref.
10	REP	VA-DLLME	6	1-octanol and ACN/0.03 and 0.1	HPLC-UV	1	10	0.40	[18]
1000	VIL, ALO, SIT, LIN, PIO, MIT, GLB, GLM	SPE	50	MeOH/5	LC-MS/MS	0.2	16	1.0	[19]
250	GLB, MET, PIO, SIT, VIL	SPE	25	MeOH/4	HPLC-Time of Flight-MS	0.7	12	0.40–32 (LOQ)	[20]
500	MET, GU, GLP, GLC, GLY, GLM	SPE	>20	MeOH/6	LC-MS/MS	0.2	9	0.0001–0.00245 (LOQ)	[21]
5	EMP, MET, SIT, GLC, REP	QuEChERS SF-μSPE	3	MeOH:ACN (80:20, *v/v*)/0.5	HPLC-UV	0.8	16	0.03–0.09	[22]
1	MET, DAP, LIR, PIO, GLC, GLM, GLA, REP	FPSE	45	ACN/0.1	HPLC-DAD	1.2	10	2.0–34.6	This study

ALO—alogliptin, LIN—linagliptin, MIT—mitiglinide, GLB—glibenclamide, GU—guanyl urea, GLP—glipizide, GLY—glyburide, EMP—empagliflozin.

## Data Availability

Data available on request due to restrictions.

## Supplementary Materials

The following supporting information can be downloaded at: https://www.mdpi.com/article/10.3390/molecules29204834/s1, Table S1. Physico-chemical properties and sources of anti-diabetic drugs used in the study; Table S2. Validation parameters of the proposed FPSE-HPLC-DAD method for the analysis of anti-diabetic drugs; Table S3. Analysis of real environmental water samples using the developed FPSE-HPLC-DAD method; Figure S1: Chemical structures of the anti-diabetic compounds used in the study; Figure S2. HPLC chromatograms of anti-diabetic drugs (1000 μg·L^−1^) detected at a 224 nm wavelength after FPSE desorbed with 100%MeOH (A) and 100%ACN (B); Figure S3. Overlay of HPLC chromatograms for the unspiked (black), spiked with 50 μg·L^−1^ (blue), and spiked with 300 μg·L^−1^ (pink) after application of the proposed FPSE-HPLC under optimized conditions at 224 nm wavelength to the lake (A), river (B), city WWTP influent (C), city WWTP effluent (D), and hospital WWTP effluent (E) water samples; Figure S4. HPLC chromatogram of anti-diabetic drugs before the optimization of HPLC conditions; Figure S5. Optimized HPLC chromatogram of anti-diabetic drugs (spiked 1000 μg·L^−1^ on Milli-Q water) separated with a Hypersil GOLD C18 column, with isocratic elution of 60:40 ACN:phosphate buffer (*v*/*v*) at 1.2 mL·min^−1^ flow rate, and detection at a 224 nm wavelength; Figure S6. SEM images of uncoated (A) and PEG-coated (B) glass microfibers, magnified 300 times, and the cross-section of PEG-coated glass microfiber at 350 (C) and 1500 (D) times magnification; Table S4. Resolution and theoretical plates before and after optimization.
