# Supplementary material for: Application of Fabric Phase Sorptive Extraction as a Green Method for the Analysis of 10 Anti-Diabetic Drugs in Environmental Water Samples
Source: Molecules. 2024 Oct 12;29(20):4834. doi: 10.3390/molecules29204834 (PMC11509965; doi:10.3390/molecules29204834)
Supplement: Supplementary file 1 [file molecules-29-04834-s001.zip › molecules-3252599-supplementary.pdf]

Application of Fabric Phase Sorptive Extraction as a Green Method for the Analysis of 10 Anti-diabetic Drugs in Environmental Water Samples

Augosto Misolas [1,2], Mohamad Sleiman [2] and Vasilios Sakkas [1,*]

[1] Department of Chemistry, School of Science, University of Ioannina, 451110 Ioannina, Greece; a.misolas@uoi.gr
[2] Institute of Chemistry of Clermont Ferrand, Sigma Clermont, UCA Campus Des Cezeaux, Université Clermont Auvergne, Clermont Auvergne INP, CNRS, ICCF, F-63000 Clermont-Ferrand, France; mohamad.sleiman@sigma-clermont.fr
* Correspondence: vsakkas@uoi.gr

Table S1. Physico-chemical properties and sources of anti-diabetic drugs used in the study

Active Pharmaceutical Ingredient	Molecular Formula	Molecular Weight (g·mol^{-1})	pKa	logK_{ow}	Anti-diabetic drug class	Brand	Formulation	Manufacturer	Address	Lot Number	Expiry
Dapagliflozin (DAP)	$C_{12}H_{25}ClO_6$	408.87	12.6	2.3	SGLT-2 inhibitor	Forxiga®	10 mg tab	AstraZeneca AB	Sweden	SL407	05-2026
Glargine (GLA)	$C_{267}H_{404}N_{72}S_6O_{78}$	6063	-	-	Insulin	Lantus® SoloStar	3.64 mg/mL solution	Sanofi Aventis	Germany	4F9423A	31-10-2026
Gliclazide (GLC)	$C_{15}H_{21}N_3O_3S$	323.41	5.8	1.5	Sulfonylurea	Diamicron®	30 mg tab	Servier	Greece	6088483	08-2026
Glimepiride (GLM)	$C_{24}H_{34}N_4O_5S$	490.62	6.2	3.9	Sulfonylurea	Dialosa®	1 mg tab	PharmaPath ABEE	Greece	307631	06-2025
Liraglutide (LIR)	$C_{172}H_{265}N_{43}O_{51}$	3751.26	9.5	-3.4	GLP-1 agonist	Victoza®	6 mg/mL solution	Novo Nordisk A/S	Denmark	PS6KT54	02-2026
Metformin (MET)	$C_4H_{11}N_5$	129.16	2.8 / 11.5	-2.6	Biguanide	Glucophage®	1000 mg tab	Petsiavas A.E.	Greece	A2707	09-2027
Pioglitazone (PIO)	$C_{19}H_{20}N_2O_3S$	356.44	5.2	3.8	Thiazolidinedione	Glitact®	15 mg tab	Win Medica A.E.	Greece	231080	04-2026
Repaglinide (REP)	$C_{27}H_{36}N_2O_4$	452.59	3.9 / 6.0	5.2	Meglitinide	NovoNorm®	1 mg tab	Novo Nordisk A/S	Denmark	NL41307	02-2027
Sitagliptin (SIT)	$C_{16}H_{15}F_6N_5O$	407.31	7.7	0.7	DPP-4 inhibitor	Sinvia®	100 mg tab	Elpen AE	Greece	STC2313D	11-2026
Vildagliptin (VIL)	$C_{17}H_{25}N_3O_2$	303.4	9.0	0.9	DPP-4 inhibitor	Galvus®	50 mg tab	Novartis Europharm	Ireland	NE9627	04-2026

Table S2. Validation parameters of the proposed FPSE-HPLC-DAD method for the analysis of anti-diabetic drugs.

**Analyte**	**LDR [1], μg·L^{-1}**	**Regression Equation**	**R^2**	**LOD, μg·L^{-1}**	**LOQ, μg·L^{-1}**	**RR % (SD)**	**Intra-day %RSD**		**Inter-day %RSD**	
							**50 μg·L^{-1}**	**300 μg·L^{-1}**	**50 μg·L^{-1}**	**300 μg·L^{-1}**
MET	20-400	y = 0.0033x + 0.0893	0.990	6.1	20.0	101.4 (2.4)	7.6	2.3	9.3	5.0
DAP	7-500	y = 0.0123x + 0.1879	0.992	2.0	6.7	99.7 (3.3)	8.6	3.3	11	5.1
LIR	50-500	y = 0.0014x + 0.0067	0.993	15.2	50.1	94.8 (6.2)	7.4	6.5	5.2	8.8
PIO	15-500	y = 0.0099x - 0.0516	0.992	4.5	14.7	97.3 (3.1)	2.7	3.2	7.7	6.9
GLC	114-500	y = 0.0043x - 0.2778	0.922	34.6	110	96.6 (5.4)	-	5.6	-	11
GLM	23-500	y = 0.0051x - 0.0115	0.993	6.8	22.5	101.1 (9.0)	5.0	8.9	9.0	11
GLA	17-500	y = 0.0053x + 0.0239	0.995	5.1	16.7	103.1 (5.5)	4.5	5.4	9.4	5.0
REP	7-500	y = 0.0163x - 0.1693	0.996	2.2	7.2	98.2 (2.5)	6.0	2.6	11	7.5

[1] LDR- Linear dynamic range

Table S3: Analysis of real environmental water samples using the developed FPSE-HPLC-DAD method

		Analytes							
		**MET**	**DAP**	**LIR**	**PIO**	**GLC**	**GLM**	**GLA**	**REP**
Lake water	Unspiked, $\mu g{\cdot}L^{-1}$ (SD)*	< LOD	< LOD	< LOD	< LOD	< LOD	14.0 (1.4)	< LOD	< LOD
	+50 $\mu g{\cdot}L^{-1}$ (SD)	56 (5.4)	35.6 (1.9)	45.7 (3.9)	50.1 (0.8)	< LOQ	42.7 (0.6)	49.4 (2.8)	47.6 (2.1)
	RR %	111.9	71.1	91.4	100.2	-	57.5	98.8	95.2
	ME %	68.4	69.6	80.4	101.7	-	60	95.1	85.7
	+300 $\mu g{\cdot}L^{-1}$ (SD)	192.9 (16.1)	253.3 (7.0)	326.8 (11.9)	258.9 (8.4)	88.2 (13)	221.9 (1.9)	287.9 (1.4)	233.5 (0.35)
	RR %	64.3	84.4	108.9	86.3	29.4	69.3	95.9	77.8
	ME %	58.2	83.4	112.8	88.5	10.9	69.7	92.7	77.9
River water	Unspiked, $\mu g{\cdot}L^{-1}$ (SD)	< LOD	< LOD	< LOD	< LOD	< LOD	19.5 (6.6)	< LOD	< LOD
	+50 $\mu g{\cdot}L^{-1}$ (SD)	28.6 (3.6)	25.9 (4.6)	22.6 (4.5)	19.3 (1.7)	< LOQ	41.6 (4.0)	34.1 (6.2)	39.6 (3.7)
	RR %	57.2	51.8	45.2	38.6	-	44.1	68.3	79.3
	ME %	34.9	48.2	43.7	31.9	-	46.0	68.2	67.4
	+300 $\mu g{\cdot}L^{-1}$ (SD)	120.4 (3.6)	186.5 (3.8)	298.7 (4.3)	108.6 (4.5)	165.6 (4.4)	226.1 (7.7)	255.4 (5.1)	244.2 (7.4)
	RR %	40.1	65.3	99.6	36.2	55.2	68.8	85.2	81.4
	ME %	36.3	60.8	103.2	36.1	45.7	69.3	82.4	81.6
City WWTP influent	Unspiked, $\mu g{\cdot}L^{-1}$ (SD)	< LOD	69.0 (1.0)	71.9 (0.4)	< LOD	< LOD	93.9 (1.3)	< LOD	< LOD
	+50 $\mu g{\cdot}L^{-1}$ (SD)	34.5 (0.9)	103.2 (1.6)	106.8 (25)	40.4 (1.8)	< LOQ	126.7 (1.4)	10.9 (2.6)	37.0 (0.5)
	RR %	69.0	68.6	69.8	80.8	-	65.5	21.8	73.9
	ME %	42.2	47.0	55.5	79.8	-	68.4	19.2	61.2
	+300 $\mu g{\cdot}L^{-1}$ (SD)	195.0 (85)	172.2 (45)	293.2 (83)	223.2 (48)	170.9 (22)	283.8 (58)	160.5 (77)	175.1 (36.4)
	RR %	65.0	34.4	73.8	74.4	57.0	63.3	53.5	58.4
	ME %	58.8	32.1	75.2	76.1	48.1	63.7	50.9	57.5
City WWTP effluent	Unspiked, $\mu g{\cdot}L^{-1}$ (SD)	< LOD	6.0 (1.5)	< LOD	< LOD	< LOD	12.6 (3.2)	< LOD	< LOD
	+50 $\mu g{\cdot}L^{-1}$ (SD)	21.1 (3.3)	22.2 (2.6)	48.0 (10.2)	40.5 (0.2)	< LOQ	60.7 (8.8)	41.7 (5.3)	47.4 (2.9)
	RR %	42.1	44.4	96.1	81.0	-	96.1	83.4	94.9
	ME %	25.7	22.3	84.1	80.0	-	100.3	81.5	85.3
	+300 $\mu g{\cdot}L^{-1}$ (SD)	126.3 (3.9)	215.4 (5.9)	287.1 (5.2)	245.4 (3.7)	82.9 (12.5)	289.2 (2.4)	266.3 (3.2)	226.5 (1.2)
	RR %	42.1	69.8	95.7	81.8	27.6	92.2	88.8	75.5
	ME %	38.1	65.0	99.3	83.8	8.5	92.7	85.8	75.5
Hospital WWTP influent	Unspiked, $\mu g{\cdot}L^{-1}$ (SD)	1158[1] (21)	< LOD	< LOD	< LOD	< LOD	28 (0.8)	< LOD	< LOD
	+50 $\mu g{\cdot}L^{-1}$ (SD)	1716[1] (82)	41.7 (3.7)	32.8 (2.2)	19.4 (1.4)	< LOQ	37.2 (0.9)	24.8 (4.8)	35.8 (1.5)
	RR %	111.7	83.4	65.6	38.9	-	18.5	49.7	71.6
	ME %	68.2	78.0	59.9	32.2	-	19.3	43.8	58.5
	+300 $\mu g{\cdot}L^{-1}$ (SD)	2793[1] (97)	189.6 (5.4)	314.7 (10)	99.8 (1.1)	177.6 (12)	228.4 (18)	292.6 (0.7)	229.6 (11)
	RR %	54.5	63.2	104.9	33.3	59.2	66.8	97.5	76.5
	ME %	49.3	63.6	108.7	33.0	51.1	67.2	92.7	76.5

Hospital WWTP effluent	Unspiked, $\mu g \cdot L^{-1}$ (SD)	392.6[2] (7.7)	< LOD	< LOD	< LOD	< LOD	< LOD	< LOD	< LOD
	+50 $\mu g \cdot L^{-1}$ (SD)	414.7[2] (38)	24.0 (0.2)	40.8 (7.6)	15.4 (0.5)	< LOQ	43.6 (2.8)	44.4 (2.0)	51.0 (1.5)
	RR %	8.8	48.0	81.6	30.8	-	87.1	88.8	101.9
	ME %	5.4	37.3	72.6	23.1	-	86.3	86.3	93.4
	+300 $\mu g \cdot L^{-1}$ (SD)	500.2[2] (4.9)	205.6 (9.4)	320.9 (20)	85.6 (2.4)	123.0 (5.8)	231.2 (15)	268.3 (17)	221.2 (12)
	RR %	7.2	68.5	107.0	28.5	41.0	77.1	89.5	73.7
	ME %	6.5	64.8	110.8	28.1	26.6	76.8	86.5	73.6

*SD: Standard Error (*N*=3)

[1] diluted 1:10 (*v/v*) with Mill-Q water

[2] diluted 2:10 (*v/v*) with Mill-Q water

Table S4. Resolution and theoretical plates before and after optimization.

Analyte	Before Optimization			After Optimization		
	Retention Time	Resolution	Theoretical Plates	Retention Time	Resolution	Theoretical Plates
MET	NA	-	-	1.407	NA	1416
VIL	NA	-	-	1.597	NA	NA
DAP	6.340	NA	7684	2.053	0.96	5657
LIR	2.197	3.11	8815	2.153	2.49	3347
GLA	2.513	6.48	8236	2.390	3.90	11237
PIO	NA	-	-	2.777	4.47	10499
GLC	3.333	4.30	8707	3.300	5.27	10940
SIT	4.073	0.85	6514	3.997	1.20	13235
GLM	4.240	8.74	7738	4.177	18.3	10554
REP	NA	-	-	8.643	NA	11214

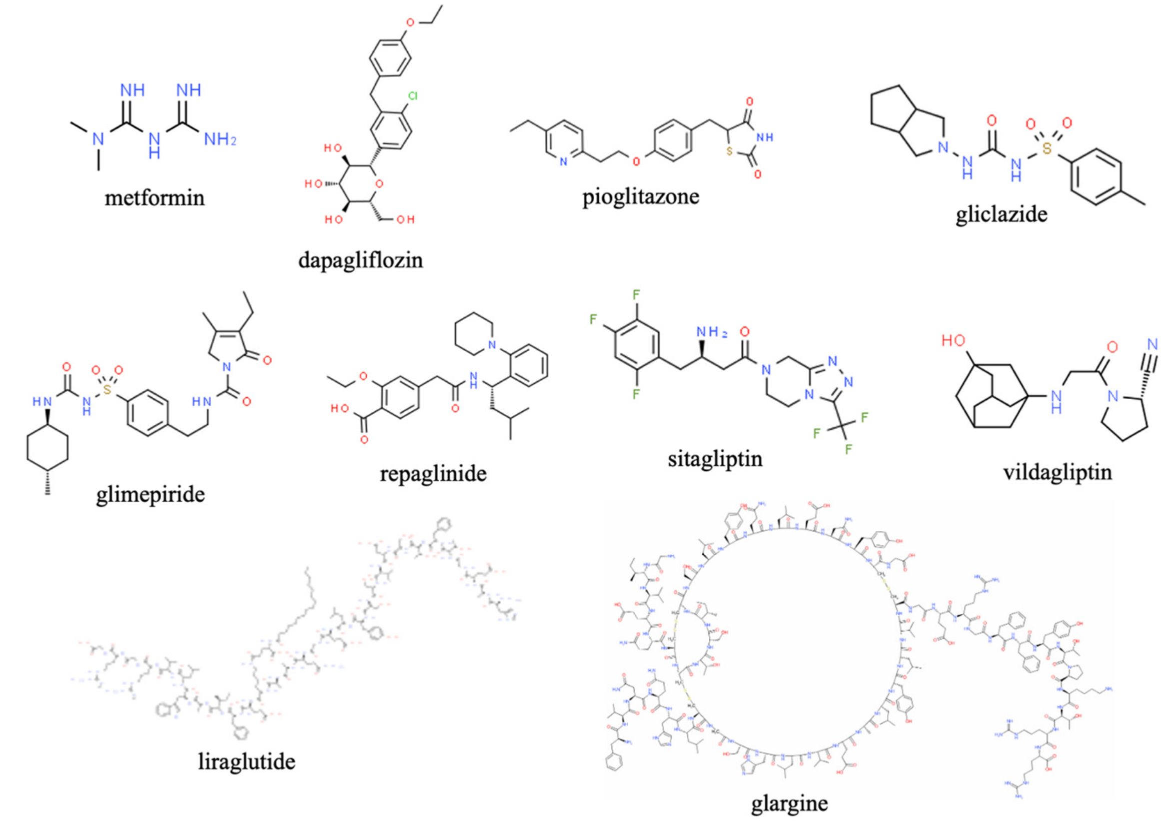

Figure S1. Chemical structures of the anti-diabetic compounds used in the study.

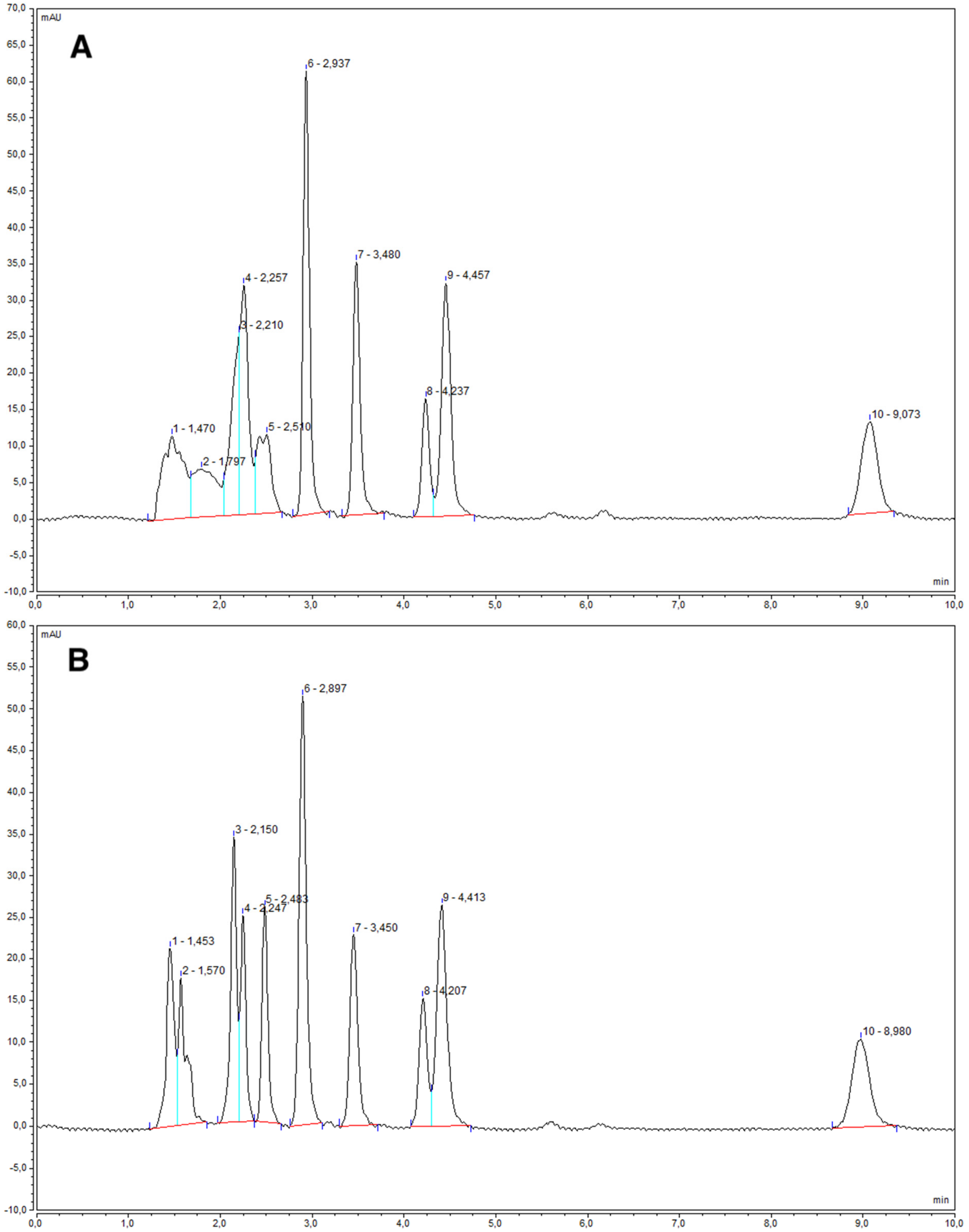

Figure S2. HPLC chromatograms of anti-diabetic drugs (1000 µg·L^{-1}) detected at 224 nm wavelength after FPSE desorbed with 100%MeOH (A) and 100%ACN (B). Peaks: 1- metformin, 2- vildagliptin, 3- dapagliflozin, 4- liraglutide, 5- glargine, 6- pioglitazone, 7- gliclazide, 8- sitagliptin, 9- glimepiride, 10- repaglinide.

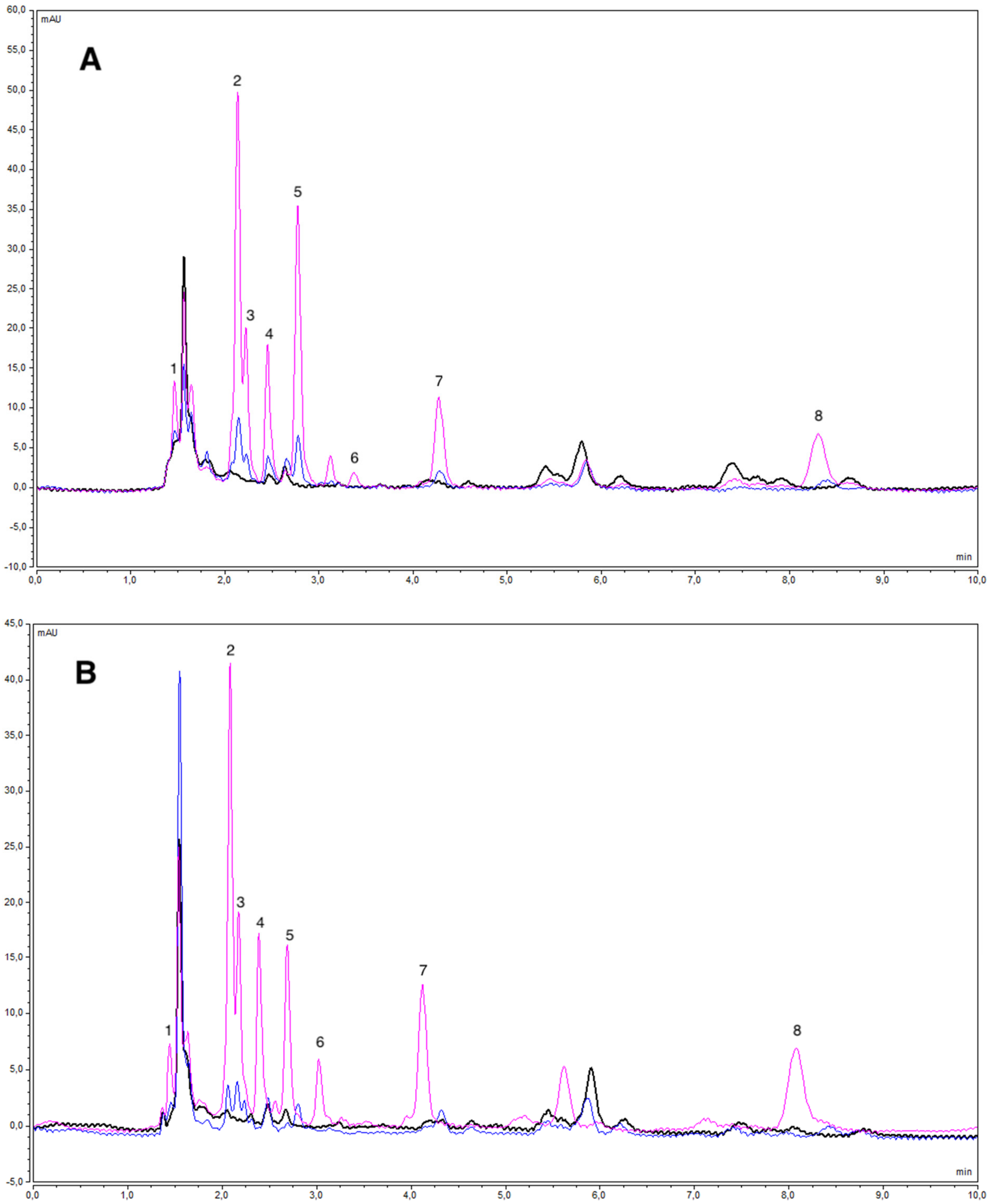
A
B
mAU
min
1
2
3
4
5
6
7
8

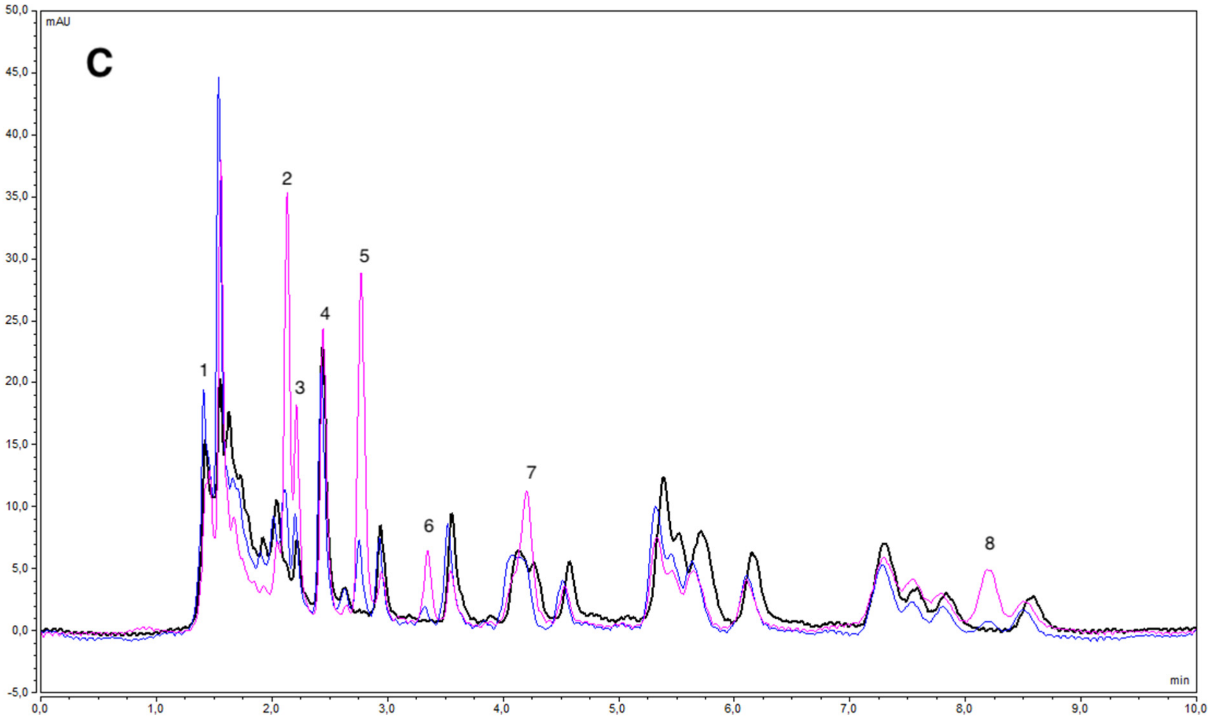
C
mAU
min
1
2
3
4
5
6
7
8
50,0
45,0
40,0
35,0
30,0
25,0
20,0
15,0
10,0
5,0
0,0
-5,0
0,0
1,0
2,0
3,0
4,0
5,0
6,0
7,0
8,0
9,0
10,0

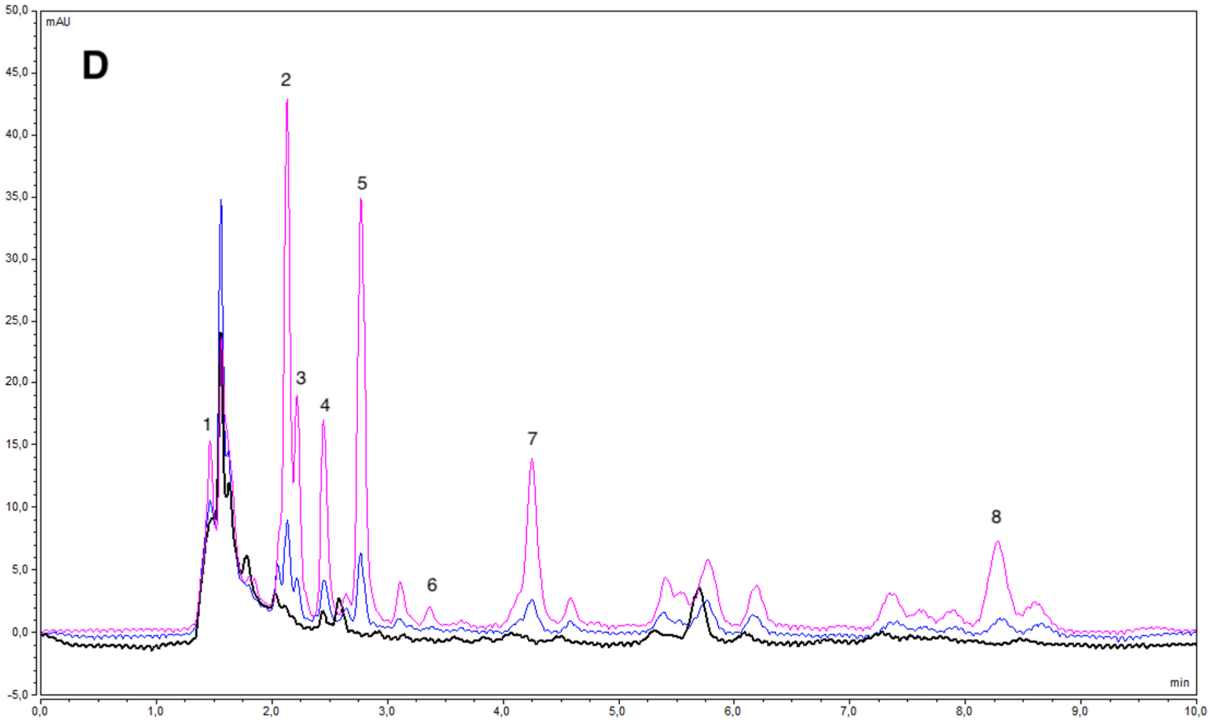
D
mAU
min
1
2
3
4
5
6
7
8
50,0
45,0
40,0
35,0
30,0
25,0
20,0
15,0
10,0
5,0
0,0
-5,0
0,0
1,0
2,0
3,0
4,0
5,0
6,0
7,0
8,0
9,0
10,0

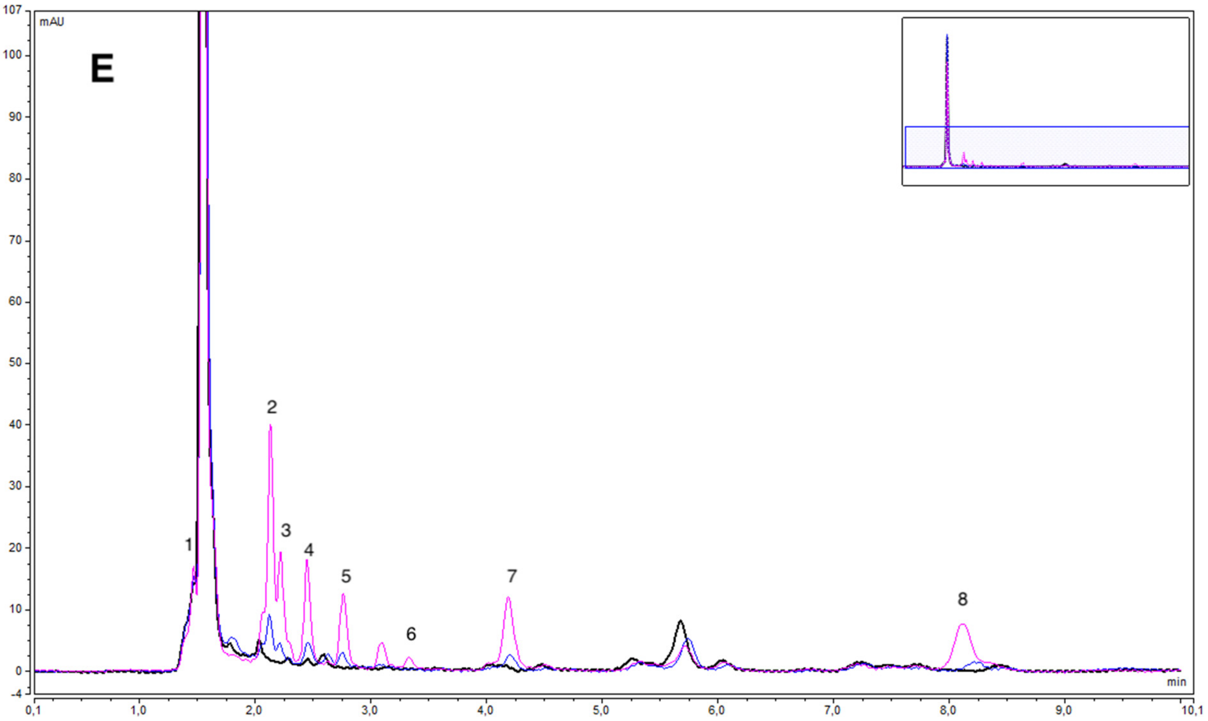

Figure S3. Overlay of HPLC chromatograms for the unspiked (black), spiked with 50 µg·L^{-1} (blue), and spiked with 300 µg·L^{-1} (pink) after application of the proposed FPSE-HPLC under optimized conditions at 224 nm wavelength to the lake (A), river (B), city WWTP influent (C), city WWTP effluent (D), and hospital WWTP effluent (E) water samples. Peaks: 1- MET, 2- DAP, 3- LIR, 4- GLA, 5- PIO, 6- GLC, 7- GLM, 8- REP.

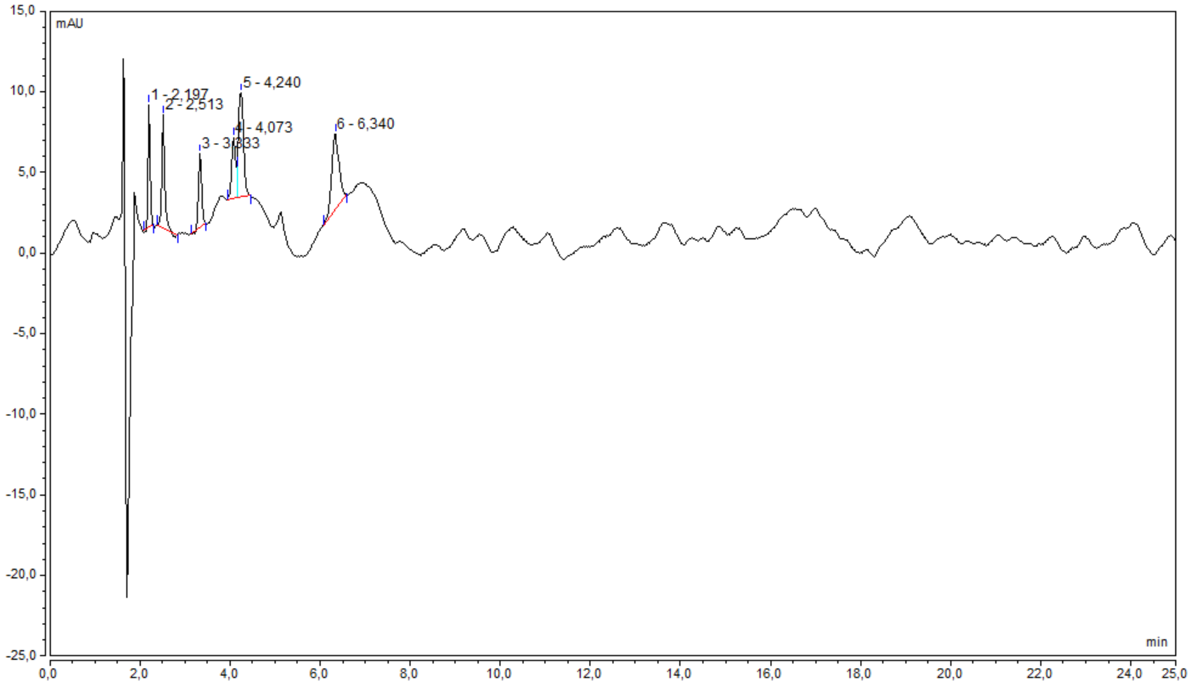

Figure S4. HPLC chromatogram of anti-diabetic drugs (spiked 1000 µg·L^{-1} on Milli-Q water) before the optimization of the HPLC conditions.

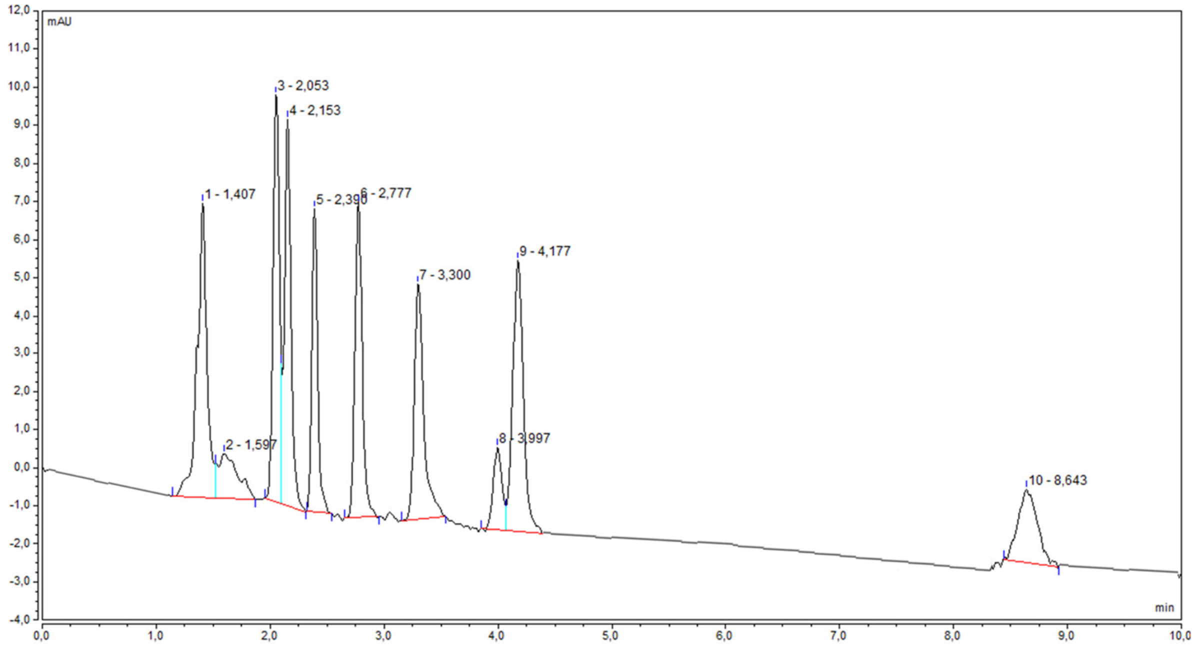

Figure S5. Optimized HPLC chromatogram of anti-diabetic drugs (spiked 1000 µg·L^{-1} on Milli-Q water) separated on Hypersil GOLD C18 column (150 × 4.6 mm), with isocratic elution of 60:40 ACN:phosphate buffer (v/v) at 1.2 mL·min^{-1} flow rate, and detection at 224 nm wavelength. Peaks: 1- metformin, 2- vildagliptin, 3- dapagliflozin, 4- liraglutide, 5- glargine, 6- pioglitazone, 7- gliclazide, 8- sitagliptin, 9- glimepiride, 10- repaglinide.

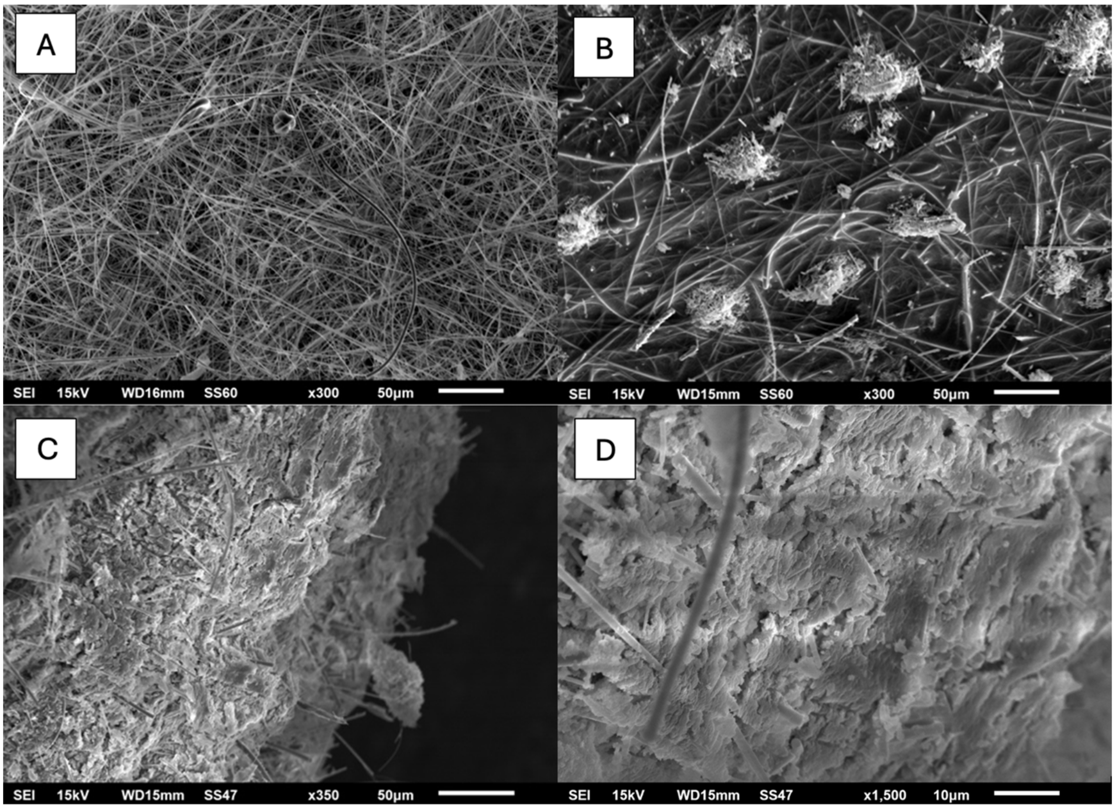

Figure S6. SEM images of uncoated (A) and PEG-coated (B) glass microfiber magnified 300 times; and the cross section of the PEG-coated glass microfiber magnified at 350 (C) and 1500 (D) times magnification.
